# Management of proximal rectus femoris injuries – do we know what we’re doing?: A systematic review

**DOI:** 10.5114/biolsport.2023.116454

**Published:** 2022-07-21

**Authors:** Lone Bogwasi, Louis Holtzhausen, Dina Christa Janse van Rensburg, Audrey Jansen van Rensburg, Tanita Botha

**Affiliations:** 1 Section Sports Medicine, Faculty of Health Sciences, University of Pretoria, Pretoria, South Africa; 2 Aspetar Orthopedic and Sports Medicine Hospital, Weill-Cornell Medical College; Qatar; 3 Department of Exercise and Sports Sciences, University of the Free State, Bloemfontein, South Africa; 4 Medical Board Member, World Netball, Manchester, UK; 5 Department of Statistics, Faculty of Natural and Agricultural Sciences, University of Pretoria, Pretoria, South Africa

**Keywords:** Rectus Femoris, Avulsions, Return, Treatment

## Abstract

Rectus femoris (RF) injury is a concern in sports. The management RF strains/tears and avulsion injuries need to be clearly outlined. A systematic review of literature on current management strategies for RF injuries, and to ascertain the efficacy thereof by the return to sport (RTS) time and re-injury rates. Literature search using Medline via PubMed, WorldCat, EMBASE, SPORTDiscus. Eligible studies were reviewed. Thirty-eight studies involving hundred and fifty-two participants were included. Majority (n = 138; 91%) were males, 80% (n = 121) sustained RF injury from kicking and 20% (n = 31) during sprinting. The myotendinous (MT), (n = 27); free tendon (FT), (n = 34), and anterior-inferior iliac spine (AIIS), (n = 91) were involved. Treatment was conservative (n = 115) or surgical (n = 37) across the subgroups. 73% (n = 27) of surgical treatments followed failed conservative treatment. The mean RTS was shorter with successful conservative treatment (MT: 1, FT: 4, AIIS avulsion: 2.9 months). Surgical RTS ranged from 2–9 months and 18 months with labral involvement. With either group, there was no re-injury within 24 months follow-up. With low certainty of evidence RF injury occurs mostly from kicking, resulting in a tear or avulsion at the FT and AIIS regions with or without a labral tear. With low certainty, findings suggest that successful conservative treatment provides a shortened RTS. Surgical treatment remains an option for failed conservative treatment of RF injuries across all subgroups. High-level studies are recommended to improve the evidence base for the treatment of this significant injury.

## INTRODUCTION

There appears to be a high incidence of muscle injuries in elite sports [1–4]. Most of these injuries occur in the lower extremities (68–88%), with 25% being indirect (non-contact) thigh muscle strains [2, 5–7]. The total time lost from activity due to muscular injury in male soccer players is estimated at around 20–37% at professional level, [1, 3, 8–14] and 18–23% at amateur level [15, 16] hence a major concern for teams and players [1, 3, 10, 17]. Quadriceps strains are prevalent in sporting codes involving kicking and repetitive sprinting actions such as soccer and rugby [2, 3, 17–19]. Ekstrand *et al.* [3] found these injuries to be the second most prevalent muscle injury in professional soccer players after hamstring injuries, leading to more missed games than other thigh injuries such as hamstring and groin injuries. A high re-injury rate (17%) is also reported [3].

The rectus femoris (RF) is a bi-articular muscle situated anteriorly on the quadriceps muscle group. It assists in knee extension, hip flexion and stabilization of the pelvis on the femur when bearing weight [20]. Its high proportion of type II fibres (65%) makes the RF highly susceptible to injury [17, 21]. A kicking injury mostly involves the thigh of the kicking leg and is frequently diagnosed as a RF muscle strain, [17, 22] often injured during eccentric loading [17, 23]. The common site of RF injuries is intramuscular strains of the musculotendinous junction [17, 23]. Serner *et al.* [22] found that a vast majority of RF injuries (94%) involved the proximal tendons, more commonly affecting the indirect tendon (56%) than the direct tendon insertion [22].

Following muscle injury there is a gradual healing process, to regain muscle strength to pre-injury level [24]. Severe re-injury may be incurred post return to sport (RTS) without optimal regain of tensile strength [25, 26]. The highest risk is the initial 2 weeks after RTS [24, 27]. Despite efforts to improve evidence on the management of muscle injuries, (British Athletics hamstring studies) [28, 29] there is still little scientific basis or evidence for the majority of the treatment protocols [30].

In the sports medicine outpatient setting the senior author has identified RF to be a common injury of concern alongside hamstring and groin injuries. Despite this, there is no standardized approach to treating RF injuries in elite athletes [31]. This is undesirable given the medical and socioeconomic burden for the injured player and the team as the safe RTS post-injury remains to be scientifically evaluated [32, 33]. The management of RF injuries has historically been either successful conservative treatment or surgical treatment following failed conservative treatment. This approach, however, remains controversial with no high-level evidence supporting either a conservative or a surgical approach as superior. Tailored evidence-based treatment approach for RF injuries would be efficient in addressing this shortfall in the sports medicine setting. It will also allow a practical and timely application of treatment principles for RF injuries to assist in successful RTS.

RF injury is subgrouped to guide on the specific anatomical management strategy. The subgroups include myofascial (MF) strains, myotendinous (MT) strains, free tendon (FT) tear and anterior inferior iliac spine (AIIS) avulsion. AIIS represents the bony origin of the direct head RF. The origins of the two RF tendons extend distally as the FT region end as the MT region where the tendon transitions into muscle. The MF injury involves any region of the RF with fascial extension [20].

The purpose of this systematic review is to report the existing best evidence on current management strategies for RF injuries based on the current concepts in management of RF injuries [20, 34]. We ascertained the efficacy of these management strategies as measured by time to RTS and re-injury rate.

## MATERIALS AND METHODS

This literature review was conducted according to the PRISMA statement [35]. The protocol was registered with the International Prospective Register for Systematic Reviews (PROSPERO, Registration number: CRD42020180962). The systematic review was conducted on CADIMA version 2.2.1, an open-access online evidence synthesis and data extraction tool that facilitates the reporting and conduction of systematic reviews.

### Literature search strategy

A comprehensive literature search was conducted electronically between 31/01/2020 and 30/06/21 using the following databases: MEDLINE via PubMed, WorldCat, EMBASE, SPORTDiscus. Combinations of the following keywords were applied, using OR / AND / NOT operators: “rectus femoris injury / anterior inferior iliac spine avulsion / sport / athlete / management / treatment”. The details of the search strategy are outlined in Supplementary Table S1.

### Study selection criteria

Study selection was performed by two independent reviewers (LB and LH). The selection criteria were as outlined below:

– Randomised control trials (RCT) and observational studies (cohort studies, case-control studies, case series, case reports) published between 1979 and 2021.
– Athletes who were diagnosed with RF injury with or without an AIIS avulsion, from a kicking or sprinting mechanism, confirmed with imaging.
– Male or female athletes (12–45 years old).
– Acute, chronic, and re-injuries.
– The management/treatment strategies outlined (i.e., conservative, or surgical).
– RTS time and/or re-injury follow-up outcome
– Articles written in English.

All studies were reviewed in parallel by title/abstract and by full text by two independent reviewers (LB and LH). Eligible studies were independently selected. Disagreement regarding the inclusion of a paper was resolved using a mediator (AS).

### Quality assessment

We ranked the level of evidence for all included studies from I to V according to the Oxford Centre for Evidence-Based Medicine (OCEBM) criteria [36] (Supplementary Table S2). The methodological quality assessment of each included study was then assessed by two authors (LB and LH) independently using a modification of the Newcastle Ottawa scale for evaluating the methodological quality of case reports and case series [37]. Each applicable question was answered “YES” or “NO” and scored as ‘high quality’ when all 5/5 criteria were fulfilled, ‘moderate quality’ when 4/5 criteria were fulfilled and ‘low quality’ when only 3/5 or fewer criteria were fulfilled [38]. Questions 4, 5 and 6 were excluded since they apply to studies involving adverse drug reactions [37]. Study assessment included (a) representativeness of the case(s), (b) ascertainment of exposure, (c) ascertainment of outcome, (d) adequacy of follow-up, and (e) adequacy of reporting. The results are available in Supplementary Table S2.

The quality assessment and a consistency check were conducted on CADIMA. Differences in individual assessments were discussed and consensus was reached between the two reviewers in all cases.

### Data extraction

The two independent reviewers conducted the data extraction process in parallel on CADIMA [39]. Additional articles from other sources were incorporated.

Article information was extracted including the study details (title, authors, year, design, and aim), participant characteristics (number of participants, age, gender, sporting activity, mechanism of injury, and diagnosis and diagnostic modalities), and treatment details (non-surgical vs. surgical), surgical indications and time from injury to surgery, time from injury to RTS (for conservative treatment), or time from surgery to RTS (for surgical treatment), and re-injury occurrence.

### Injury classification

Based on reported details in the studies, the RF injuries were divided into four subgroups: [40, 41] 1) myofascial (MF) injuries, 2) myotendinous (MT) injuries, 3) free tendon (FT) injuries, and 4) anterior inferior iliac spine (AIIS) avulsion fractures. Injury combinations were also reported if it was described like that in an included study.

## RESULTS

### Literature search

**FIG. 1:**
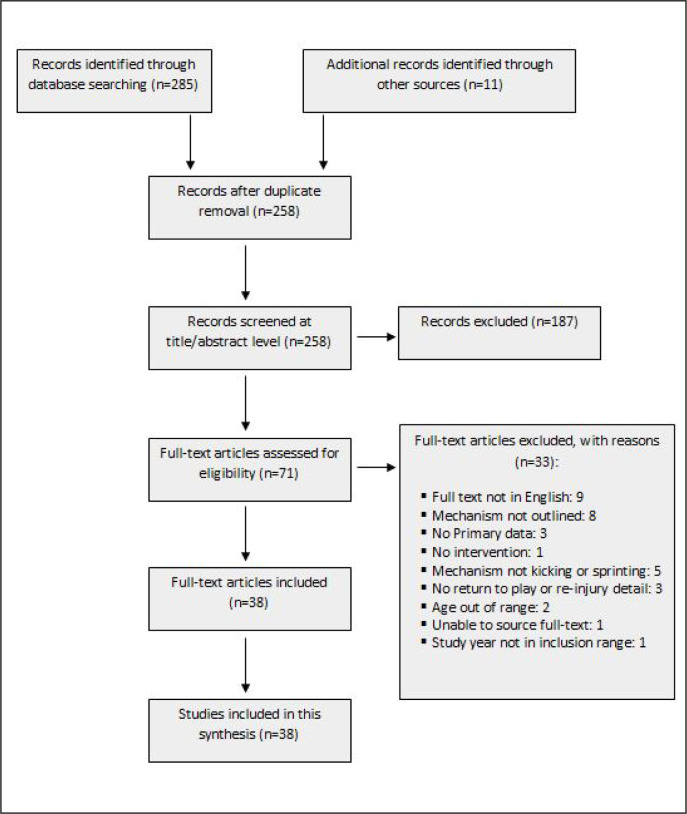
Flow diagram of the study selection process.

The initial database search yielded 285 records and a further 11 records were identified from other sources. Duplicates were removed and the title and abstracts of 258 studies were evaluated. We excluded 187 records based on study selection criteria, leaving 71 records for full-text evaluation. After a full-text review, 38 studies were included. Reasons for exclusion following full-text review are listed in Supplementary Table S3, and a flow diagram of the study selection process is presented in Figure 1. A consistency check on the eligibility criteria application by both reviewers yielded a Kappa value of 0.94, which is considered ‘excellent agreement’. Inclusion disagreements were settled by discussion and consensus with the moderator (AS) in seven studies.

### Description of included studies

#### Study design & quality

Of the 38 included studies, 13 were case series and 25 were case reports, and all studies were ranked at an OCEBM level of IV. Assessment of methodological quality revealed fourteen of the studies as high quality (36.8%), [21, 42–51] six as moderate quality (15.8%) [52–57] and eighteen as low quality (47.4%) [58–74] (Supplementary Table S2). The risk of bias is inverse to the study quality.

#### Study participants

From 38 studies 152 participants were included. The majority (n = 138; 91%) was males sustaining RF injuries during either soccer, American football, athletics, softball, baseball, or rugby (Table 1).

**TABLE 1:** Summary of studies included.

Injury type (Number of studies)	Number of athletes (n)	Type of sport (n)	MOI (n)	Diagnostic Modality (n)	Intervention (n)	Primary outcome measure (RTS time, Re-injury)
Myotendinous [48, 58, 68, 69, 72, 77, 78] (n = 7)	27	Soccer (20) American football (3) Softball (2) Baseball (2)	Kicking (17) Sprinting (10)	MRI (15) US (12)	Conservative (18) Surgical (9)	RTS: Conservative: 1 month (4 weeks) Surgical: 6 (2–9) months Re-injury: None in 24 months

Free tendon [21, 43–45, 49, 51, 61–63, 79] (n = 10)	34	Soccer (20) American football (12) Athletics – Track (2)	Kicking (21) Sprinting (13)	US (1) MRI (30) MRA (3)	Conservative (17) Surgical (17)	RTS: Conservative: mean 14.5 (4–36) weeks Surgical: 4.6 (2–9) months – Surgery as first line: 3 months – Surgery (failed conservative): 5 months Re-injury: None between 2 and 35 months follow up

Avulsion fracture [42, 46, 47, 49, 50, 52–57, 64–67, 71, 73–76, 80, 81] (n = 23)	91	Soccer (26) Athletics (6) Rugby (1) Fighting sport (1) Gym (1) Not specified (56)	Kicking (83) Sprinting (8)	X-ray (69) CT Scan (7) MRI (15)	Conservative (80) Surgical (11)	RTS: Conservative: 2.9 (1.5–9) months Surgical: 6 (1.5–18) months Re-injury: None between 12 and 24 months follow up

No isolated myofascial injuries were reported in the included studies. n = number of athletes; MOI = Mechanism of injury; MRA = Magnetic resonance arthrogram; MRI = Magnetic resonance imaging; US = Ultra sound; CT = Computerized tomography scan; RTS = Return to sport.

### Summary of injury detail of participants

Amongst the 152 participants, 80% (n = 121) sustained RF injury as a result of kicking [21, 43–53, 55, 58, 60, 61, 63–69, 71–73, 75–77] and the remaining 20% (n = 31) were injured during sprinting [21, 42, 49, 54, 56, 57, 60–62, 74, 75, 77].

Following the RF injury classification model, [40, 41] the mechanism of injury, diagnostic modality, intervention, and primary outcome measure for each RF injury sub-group (MF, MT, FT and AIIS avulsion) amongst the included studies are described in Table 1.

### Myofascial injuries

No isolated MF injuries were reported in the included studies. All MF injuries reported were combined injuries with a labral tear and either a MT, [58] FT or AIIS injury [49, 63].

### Myotendinous injuries

Twenty-seven athletes in seven studies suffered a MT injury [48, 58, 68, 69, 72, 77, 78] mainly sustained from playing soccer (74%), and American football (19%).

***Mechanism of injury:*** A variety of MT injury patterns relates to kicking, with 88% resulting in a tear at either the central aponeurosis (n = 4) [48] or MT junction (n = 11) [68, 69, 72, 78]. All MT injuries from the sprinting group occurred at the MT junction [77].

***Diagnostic modality:*** All athletes were evaluated using either a Magnetic Resonance Imaging (MRI) [48, 58, 60, 68, 69] or an ultrasound (US) scan [72, 78].

***Intervention:*** Conservative approach or surgical treatments were reported for MT injuries but no standardized management amongst the studies was reported (Table 2).

**TABLE 2:** Details of myotendinous (MT) treatment per study.

MT INJURIES CONSERVATIVE TREATMENT					

AUTHOR, YEAR	NUMBER OF ATHLETES	MECHANISM	INJURY DESCRIPTION	CONSERVATIVE PROGRAM DETAIL	RTS, RE-INJURY
Patel *et al*, 2019 [72]	1	Kicking	RF mid-substance incomplete partial thickness tear	Non-steroidal anti-inflammatory drugs and hip flexor and quads stretching exercises; PRP at 6 weeks plus an eccentric program	RTS: 4 weeks (1 month) post PRP Re-injury: NR

Hughes *et al*, 1995 [77]	6	Sprinting	Proximal RF Incomplete tear, intrasubstance strain	Not outlined	RTS: NR Re-injury follow up: no limitation at 18–36 months

Fermín Valera-Garrido *et al*, 2020 [78]	11	Sprinting: 3 Kicking: 8	Grade II RF injury at the MT junction.	US-Guided Percutaneous Needle Electrolysis (PNE). The PNE technique was performed under ultrasound guidance on the muscle injury using an intensity of 1.5–2 mA during 3 s, five times (1.5–2:3:5), according to the protocol by Valera-Garrido and Minaya-Muñoz followed by a specific rehab and reconditioning program.	RTS: 15.62 ± 1.80 days Re-injury: No injury at 5 months (20 weeks)

**Table:** 

MT INJURIES SURGICAL TREATMENT							

AUTHOR, YEAR	NUMBER OF ATHLETES	MECHANISM	INJURY DESCRIPTION	OPERATION INDICATION	DELAY TO SURGERY	SURGICAL TECHNIQUE	RTS, RE-INJURY
Wittstein, 2011 [48]	4	Kicking	RF Central aponeurosis tear	Failed conservative (scar tissue)	12 (6–24) months	Failed conservative (scar tissue)	Mean RTS: 9 (7–12) months; Re-injury: At 12 months, two symptomatic players at 1 and 2 years respectively; both subsided with NSAIDs, manual therapy and rehabilitation

El-Husseiny *et al*, 2012 [58]	1	Kicking	RF tear (with calcification) and labral tear	Failed conservative treatment (calcification, labral tear)	8 months	Arthroscopic excision of calcification and labral repair	RTS: 8 weeks Re-injury follow up: NR

Hughes *et al,* 1995 [77]	2	Kicking: 1 Sprinting: 1	Proximal RF Incomplete tear, intrasubstance strain	Failed conservative treatment (fibrosis)	5 and 39 months respectively	Fibrous tissue excision	RTS: NR Re-injury follow up: no limitation at 18–36 months

Straw *et al*, 2003 [68]	1	Kicking	Rupture of the rectus femoris muscle at the proximal MT junction	Failed conservative treatment	12 months	Muscle suturing to proximal tendon	RTS: 6 months Re-injury follow-up: NR

Taylor, 2012 [69]	1	Kicking	Rectus femoris muscle belly tear	Failed conservative (scar tissue)	15 months	Scar tissue release and Kessler suture of torn muscle ends with an artificial LARS system	RTS: 8 months, Re-injury follow up: NR

MT = Myotendinous; FT = Free tendon; AIIS = Anterior inferior iliac spine; PRP = platelet-rich plasma injection; NR = Re-injury follow up not reported; RTS = Return to sport.

Three studies (n = 18) reported conservative management [72, 77, 78]. In these athletes the majority sustained a form of MT tear during kicking (66%, n = 12). The details of the conservative program reported varied with either an eccentric program [72] or an US-Guided Percutaneous Needle Electrolysis (PNE) protocol [78] (Table 2).

There were five studies reporting on athletes (n = 9) with MT injuries who underwent surgical intervention [48, 58, 68, 69, 77]. The main mechanism of injury was kicking (89%, n = 8) and they sustained some form of MT tear [3, 6, 7, 68]. The indications for surgical intervention in all athletes were failed conservative treatment (Table 2).

The delay to surgery varied between 5–39 months (Table 2). The intra-operative findings were fibrosis or scar tissue formation, and surgical interventions mainly included scar tissue/fibrosis excision (n = 7). One athlete with a calcification underwent arthroscopic excision [58].

***Primary outcome measure (RTS, re-injury):*** The primary outcome was gauged by the RTS period and/or the re-injury rate of either intervention. The RTS, re-injury and follow-up reporting were inconsistent throughout the studies. (Table 2).

Athletes treated conservatively RTS within four weeks after either post-platelet-rich plasma (PRP) injection [72] or US-Guided PNE protocol [78]. No re-injury detail was reported in the PRP group [72] and in the US-Guided PNE group [78] there was no re-injury at 5 months follow-up. Hughes *et al.* [77] reported on six athletes treated conservatively in the MT injury group, the RTS outcome was not outlined. No re-injury was reported at their 18–36 months follow-up appointments inclusive of those that had surgical intervention (n = 2) [77].

In four of the five studies (n = 7) on athletes treated surgically; a mean RTS at 6 (2–9) months was reported [48, 58, 68, 69]. In three of these athletes there was no report on re-injuries [58, 68, 69]. In the other four athletes there were no re-injuries at 12 months follow-up [48].

### Free tendon

The FT tear group involved 34 athletes from ten studies [21, 43–45, 49, 51, 61–63, 79]. FT injury may involve the direct and indirect tendon individually or both at the same time [49, 63]. This may be with or without involvement of the conjoint tendon, [51] MT or MF structures [49].

***Mechanism of injury:*** Most of the injuries sustained were from kicking (64%, n = 22) playing American football or soccer [21, 43–45, 49, 51, 61, 63, 79].

Isolated FT tears were reported in 82% (n = 27/34) of athletes from six studies [21, 43–45, 49, 61, 62]. Isolated direct tendon presented as the common culprit (71%, n = 24/34) and the majority of them resulted from kicking (58%, n = 14/24) [21, 43–45, 61, 62]. In four studies of the remaining athletes (n = 10), four sustained an indirect tendon injury; [49, 79] of which three had a tear with partial-thickness labral tears [49]. Two athletes had both a direct and indirect tendon tear during kicking and sprinting respectively [49, 63] while four sustained a tear at the conjoint tendon from a kicking mechanism [51].

***Diagnostic modality:*** Most athletes were evaluated using MRI as the diagnostic modality. Magnetic resonance arthrogram (MRA) was used to diagnose 3 athletes [49] while only one athlete was diagnosed with an US scan [63].

***Intervention:*** Conservative (n = 17) [43, 49, 61, 63] and surgical interventions (n = 16) [21, 44, 45, 51, 62] were reported in four and five studies respectively in athletes with FT injuries.

Most athletes that underwent conservative treatment sustained the injury during sprinting (59%, n = 10/17) [49, 61]. The rehabilitation programs were not outlined and the duration of the rehabilitation programs was not clearly specified [61]. The treatment protocols are summarized in Table 3.

**TABLE 3:** Details of free tendon (FT) treatment per study.

FT INJURIES CONSERVATIVE TREATMENT					

AUTHOR, YEAR	NUMBER OF ATHLETES	MECHANISM	INJURY DESCRIPTION	CONSERVATIVE PROGRAM DETAIL	RTS, RE-INJURY
Hsu *et al*, 2005 [43]	2	Kicking	Direct head	Isokinetic and isometric strengthening	RTS: 4 weeks (1 month) Re-injury: None at 8 months and 3 years respectively

Foote *et al*, 2013 [49]	4	Kicking: 2 Sprinting: 2	Indirect free tendon tear with labral tear (3) Direct and Indirect avulsion (1)	Rest followed by controlled rehabilitation	RTS: 36 weeks (9 months) No re-injury at 15 months

Gamradt *et al*, 2021 [61]	10	Kicking: 2 Sprinting: 8	Direct RF	Not outlined	Mean RTS: 69.2 (21–208) days i.e., mean 10 weeks Re-injury follow up: Not reported

Esser *et al*, 2015 [63]	1	Kicking	Direct and Indirect RF	Core, pelvic, thigh strength rehabilitation	RTS: 8 weeks (2 months) Re-injury follow up: Not reported

**Table:** 

FT INJURIES SURGICAL TREATMENT							

AUTHOR, YEAR	NUMBER OF ATHLETES	MECHANISM	INJURY DESCRIPTION	OPERATION INDICATION	DELAY TO SURGERY	SURGICAL TECHNIQUE	RTS, RE-INJURY
Bottoni *et al*, 2009 [62]	1	Sprinting	RF avulsion without AIIS avulsion (FREE TENDON-DIRECT)	Direct tendon avulsion – Patient preference	NR	Fibre-wire locking stitch and bone tunnel fixation to AIIS	4 months, NR

Adler *et al*, 2014 [44]	1	Kicking	RF complete avulsion without AIIS involvement – chronic (FREE TENDON-DIRECT)	Failed Conservative (scar tissue)	15 months	Direct head re-contour over superior acetabular ridge with PEEK Corkscrew anchor	6 months, No injuries between 8 and 12 weeks follow up

García *et al*, 2011 [45]	5	Kicking	Proximal RF ruptures – FREE TENDON – DIRECT	Direct tendon avulsion – Done as first line of management	9.1 days (5–14 days)	Kranckow stitches × 3, suture anchor repair plus Intra-op direct PRP – Post-op NWB 3–4 weeks – Eccentric train @5–6 weeks	2.5 months, No re-injuries at 35 (13–63) months follow up

Irmola *et al*, 2007 [21]	5	Kicking: 4 Sprinting: 1	Proximal avulsion of RF (no AIIS involvement) – (FREE TENDON-DIRECT)	Failed conservative (3–4 cm retraction of avulsed free tendon)	53 (18 to 102) days	Suture anchor repair	9 (5–10) months, No re-injuries reported at 20 (9–45) months follow-up

Ueblacker *et al*, 2015 [51]	4	Kicking	Proximal RF avulsion without bony involvement (FREE TENDON-CONJOIN	First line management: > 2 cm retraction of avulsed segment	60 +/- 88 days	Suture anchor repair of proximal RF avulsion	4 (3–5) months, No re-injuries at 35 +/- 6 months

Huri *et al*, 2014 [79]	1	Kicking	RF indirect tendon free tendon avulsion	Failed conservative (scar tissue)	24 months	Suture anchor repair with PDS (poly-dioxanone) sutures	2 months No re-injury at 6 & 24 months

MT = Myotendinous; FT = Free tendon; AIIS = Anterior inferior iliac spine; PRP = platelet-rich plasma injection; NR = Re-injury follow up not reported; RTS = Return to sport.

The surgical techniques and indications vary amongst studies. All studies (n = 17) reporting surgical treatment employed a core principle of re-anchoring the torn tendon to its origin. The athletes included fifteen kickers and two sprinters [21, 44, 45, 51, 62, 79]. The details of the surgical techniques are described in Table 3.

Garcıá *et al*. [45] conducted surgical intervention on five athletes immediately after injury (delay to surgery: mean 9.1 (5–14) days post-injury). The surgical indications in other studies varied and amongst them included patient preference (n = 1), [62] > 2 cm tendon retraction on MRI (n = 4) [51] and a failed conservative treatment program (n = 7) [21, 44, 79]. Scar tissue formation was found in most athletes from the failed conservative treatment program [44, 79].

***Primary outcome measure (RTS, re-injury):*** The mean RTS time was 14.5 (4–36) weeks for the conservative treatment group [43, 61, 63]. The RTS time was longer at 36 weeks in three athletes treated conservatively with indirect tendon avulsions involving the labrum. No conservatively treated athletes had a re-injury on follow-up [43, 49]. Two studies (n = 11) did not present re-injury follow-up [61, 63].

The mean RTS period after surgery was 5 (2–9) months in all studies for all injury patterns and surgical techniques used, as noted in Table 3 [21, 44, 45, 51, 62, 79]. The RTS time was less than 6 months when surgery was performed acutely [45, 51, 62] and longer with surgery after failed conservative therapy [21, 44]. Where reported there were no injuries noted in the re-injury follow-up periods ranging between 2 and 35 months [21, 44, 45, 51, 79].

### Avulsion fracture of rectus femoris insertion on AIIS

Ninety-one athletes sustained a RF injury with an AIIS avulsion fracture in twenty three studies included in this systematic review [42, 46, 47, 49, 50, 52–57, 64–67, 71, 73–76, 80, 81].

***Mechanism of injury:*** Over 90% of the avulsions (n = 83/91) were sustained during kicking [46, 47, 49, 50, 52, 53, 55, 64–67, 71, 73, 75, 76, 80] and the remainder occurred during sprinting [42, 54, 56, 57, 74, 75, 81]. The majority of these injuries (95%) occurred in adolescent males (n = 87). In 61% (n = 56/91) of the athletes the specific sport was not outlined, [71] and where reported most RF injuries were sustained during kicking in soccer players (29%, n = 26) [46, 47, 49, 50, 52, 53, 55, 64–67, 73, 75, 76, 80].

***Diagnostic modality:*** RF avulsion injuries in nine studies were diagnosed on plain X-rays only (n = 69) [49, 55–57, 65, 71, 73, 74, 76, 80, 81]. In three studies computed tomography (CT scan) was used for further evaluation (n = 7), [53, 66, 67] while MRI was the investigation modality of choice for athletes in the remaining studies (n = 15) [42, 46, 47, 50, 52, 54, 64, 75].

***Intervention:*** Conservative treatment was used in most athletes (87.9%, n = 80) [49, 53, 56, 57, 64–66, 71, 74–76] of which the majority (70%) were adolescents in one pooled study on avulsion fractures [71]. The conservative program details in this particular study were however not outlined [71]. The conservative treatments per study are outlined in Table 4.

**TABLE 4:** Details of anterior inferior iliac spine (AIIS) treatment per study.

AIIS AVULSION CONSERVATIVE TREATMENT					

AUTHOR, YEAR	NUMBER OF ATHLETES	MECHANISM	INJURY DESCRIPTION	CONSERVATIVE PROGRAM DETAIL	RTS, RE-INJURY
Foote *et al,* 2013 [49]	1	Kicking	Avulsion of reflected RF /Indirect head with partial labral tear	Conservative (Controlled Rehabilitation)	RTS: 9 months Re-injury follow-up: No injury at 15 months follow up

Atalar *et al,* 2007 [53]	1	Kicking	AIIS Avulsion	Conservative (NSAIDs, rehabilitation)	RTS: 8 weeks (2 months) Re-injury follow up: Not reported

Yildiz *et al,* 2005 [56]	1	Sprinting	Bilateral (Sequential) avulsion fracture of the AIIS, occurring first in the right and then the left AIIS (4 months apart).	Conservative (gradual rehabilitation program) – both sides	RTS: not detailed, Re-injury follow up: No injury at 2 year follow up

Mader, 1990 [57]	1	Sprinting	AIIS Avulsion	Conservative (2 days complete strict bedrest, progression to full weight-bearing as symptoms improved)	RTS: 6 weeks (1.5 months) No re-injury at 6 months

Pogliacomi *et al,* 2019 [64]	1	Kicking	RF MT junction rupture AIIS avulsion	Conservative: (× 3 ten-day interval Serial PRP ultrasound-guided injections and a specific rehabilitation protocol)	RTS: 90 days (12 weeks), Re-injury follow up: Not reported

Reina *et al,* 2010 [65]	2	Kicking	AIIS avulsion fracture (chronic and acute respectively)	Conservative (physiotherapy program)	RTS: 2 and 3 months respectively, Re-injury: Not reported

Serbest *et al,* 2015 [66]	4	Sprinting	AIIS avulsion fractures	Conservative (rest, NSAIDs, gradual rehabilitation)	RTS at 10 weeks, Re-injury follow up: not reported

Schuett *et al,* 2015 [71]	56	Kicking	AIIS avulsion	Not outlined	RTS: Not outlined Re-injury: 1 non-union (referred for ORIF – declined), 25 hip pains, and 8 multiple injuries at 4-month follow-up.

Gomez, 1996 [74]	1	Sprinting	Bilateral AIIS avulsion fractures	Conservative (ICE pack, NSAIDs, limited weight-bearing – 7 days. Followed by Full weight-bearing, range of motion exercises including hip extension, hip flexor strength and conditioning on stationary bicycle.	RTS at 10 weeks Re-injury: None at 3 months follow up

Uzun *et al,* 2014 [75]	9	Kicking: 6 Sprinting: 3	AIIS avulsion fractures	Not outlined	RTS: not reported, No re-injury at 24 months

Aksoy *et al,* 2014 [76]	1	Kicking	Avulsion fracture of the right AIIS	Conservative (gradual rehabilitation program)	RTS: 12 weeks, Re-injury follow up: not outlined

Lasky-McFarlin *et al,* 2020 [80]	1	Kicking	Bilateral AIIS avulsion fractures: an acute fracture on the right and healing fracture on the left.	Conservative (The 5-phase rehabilitation protocol outlined by Metzmaker and Papas)	RTS: 8 weeks Re-injury: nil at 20 weeks

Cameron & Wallace, 2020 [81]	1	Sprinting	Asynchronous bilateral AIIS apophyseal avulsion fractures	Conservative (Rest, activity modification)	RTS: 3 months Re-injury: not outlined

**Table:** 

RF AIIS AVULSION FRACTURE SURGICAL DETAILS								

AUTHOR, YEAR	NUMBER OF ATHLETES	MECHANISM	OPERATION INDICATION	DELAY TO SURGERY	SURGICAL TECHNIQUE	INTRAOPERATIVE INJURY DESCRIPTION	RTS	RE-INJURY
Nakano *et al,* 2018 [42]	1	Sprinting	Failed conservative (physiotherapy): extra-articular anterior hip impingement (after 6 months)	6 months	Arthroscopic excision	AIIS avulsion fracture	2 months	Nil at 24 months

Matsuda *et al,* 2012 [54]	1	Sprinting	Failed conservative (rest, activity modification, NSAIDs): mal-union of AIIS (after 3 months)	9 months	Arthroscopic: fluoroscopic templating with excision of mal-united AIIS and labral re-fixation	Direct head avulsion fracture with non-union at the AIIS and surrounding callus formation	18 months	NR

Milankov *et al,* 2011 [47]	1	Kicking	Failed conservative (rest, RTS with persistent pain, underwent initial op at 4 months), – underwent excision	4 months	Modified Smith – Petersen approach: Excision of avulsed AIIS segment	Bony protuberance at the adjacent left superior rim with fraying of the labrum with RF fibers attached. No pseudo-arthrosis	4 months	Complication 2 years post-op: (heterotopic ossification in vastus intermedius tendon and its femoral insertion) – MRI showed heterotopic ossification with was excised via a modified Smith – Petersen approach: RTS = 4 months

Foote *et al,* 2013 [49]	1	Kicking	Failed Conservative (Physiotherapy, controlled rehabilitation) 6 months	6 months	Arthroscopic: debridement, labral repair and synovectomy	Pt 1: A mass of exuberant callus around the left AIIS Pt 2: Right AIIS avulsion 2.5cm distally	7 months	NR

Scillia *et al,* 2017 [50]	1	Kicking	Failed conservative (physical therapy, acupuncture) after 12 months	12 months	Modified Smith Petersen approach: excision of avulsed segment and suture anchor repair and bone wax application	avulsion of both heads of the RF and a chondrolabral separation	6 months	Nil at 18 months

Alhaneedi *et al,* 2015 [52]	1	Kicking	Failed conservative treatment (NSAIDs, physiotherapy): after 3 months	24 months	Surgical excision: anterior hip approach	Well corticated heterotrophic ossification in the right RF near the AIIS	1.5 months	Nil at 12 months

Rajasekhar *et al,* 2000 [55]	2	Kicking	Both Failed conservative: Pt 1 = (rest, analgesia, physiotherapy): exuberant callus removed at 2 years AND Pt 2 = rest and analgesia with surgery acutely).	2 and 0 years	Pt 1: Modified Smith Petersen approach; A mass of exuberant callus was excised Pt 2: Re-fixation of avulsed fracture with a 6.5mm screw and washer	Right AIIS avulsion	NR	NIL in both at 18 and 12 months

Carr II *et al*, 2017 [46]	1	Kicking	Failed conservative (rest, 2 rounds of gradual therapy): after 5 months	18 months	Anterior Smith-Petersen approach: Excision of 8mm callus and avulsed segment, sub-spinal decompression	Peripheral focal synovitis at the level of AIIS with mal-union, superolateral labral tear	5 months	Nil at 12 months

Shibahara *et al,* 2017 [67]	1	Kicking	Failed conservative: Non-union of AIIS and antero-superior labral tear after 6 months	4 months	Arthroscopic: labral repair, AIIS decompression with motorized round burr and femoroplasty	Hypertrophic mal-united bony fragment at the Right AIIS	4 months	NR

Saluan et al, 1997 [73]	1	Kicking	Failed conservative (rest, ice)	11 months	Modified Smith Petersen approach: scar tissue excision and re-fixation of avulsed segment	Avulsion of AIIS	6.5 months	Re-injury: not reported

MT = Myotendinous; FT = Free tendon; AIIS = Anterior inferior iliac spine; PRP = platelet-rich plasma injection; NR = Re-injury follow up not reported; RTS = Return to sport.

Surgical treatment was conducted in 10 studies (n = 11) and in all cases, it was done after a failed conservative treatment program. The majority (81%, n = 9) of these were from kicking injuries [42, 46, 47, 49, 50, 52, 54, 55, 67, 71, 73]. The surgical indications included non-union, [46, 49, 50, 67] mal-union [52, 54] and heterotrophic ossification [42, 47, 52]. In some of these cases there was a secondary impingement that warranted surgical correction [49, 52, 54, 67]. The delay to surgical intervention ranged between 2 and 24 months.

Table 4 shows the surgical details for the reported studies. The surgical approach was either a Smith-Petersen approach (n = 7) [46, 47, 50, 52, 55] or arthroscopic (n = 4) [42, 49, 54, 67]. The procedure was mainly an open reduction and internal screw fixation (ORIF) (n = 5) [46, 50, 55, 73] or surgical excision of the avulsed segment (n = 4) [42, 47, 52, 54].

***Primary outcome measure (RTS, re-injury):*** The RTS and re-injury rates were poorly reported for AIIS avulsion injuries, with most studies only reporting one of the two. The RTS period amongst the conservative treatment group was not outlined in three studies (n = 66), [56, 71, 75] however, ranged between 1.5 and 9 months (mean 2.9 months) in the reported studies (n = 14) as noted in Table 4. In most studies, no re-injury follow-up was reported, and in four studies involving twelve athletes, no re-injuries were reported on follow-up appointments at specifically 3, [74] 6, [57] 20 [80] and 24 months [56, 75] respectively (Table 4).

The mean RTS period for all the surgically treated athletes was 6 months (1.5–18 months), (Table 4). Matsuda *et al.* [54] reported a longer RTS time of 18 months due to AIIS avulsion fracture complicated by non-union. Studies that reported on re-injury did not have any injuries in athletes between 1- and 2-year follow-up period [42, 46, 50, 52, 55].

The modified Harris Hip Score and Non-arthritic Hip Score were used in two studies as a measure to gauge post-operative outcome [50, 67]. Both athletes scored 100 on follow-up, of which one athlete’s pre-operative score gauged improvement post-intervention [67]. Objective outcome measures were not reported in any other studies.

## DISCUSSION

The RF is the most commonly injured muscle in the quadriceps group. RF injuries have longer RTS times than hamstring and groin injuries and have a high re-injury rate [82–84]. This is the first systematic review reporting on RF injury management. The level of evidence in all 38 included studies is low with all studies being either case reports or case series. Surprisingly this indicates that although a significant injury, not much evidence-based research regarding the treatment is available. It is further supported by the heterogeneity of treatment approaches and evidenced by the moderate and low methodological quality reported in most studies e.g., treatment protocols differ, RTS not reported, re-injury not reported. The synthesis therefore points to a low certainty of evidence on the findings.

**FIG. 2:**
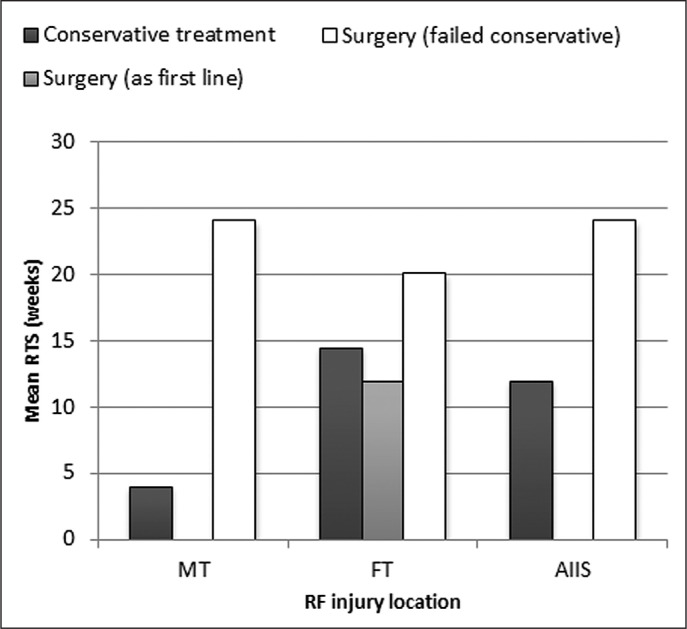
Rectus femoris (RF) injury treatment outcomes.

The sub grouped analysis based on the anatomical location allows for decision making around recovery or prognosis in the form of RTS and re-injuries [85]. The injury location and characteristics from imaging provide the anatomical site, percentage of cross-sectional involvement or presence of peri-fascial fluid. These findings provide guidance during the rehabilitation period e.g. proximal tendon injuries with > 50% cross-sectional involvement and pre-fascial fluid, are associated with a longer RTS [20]. The implication is that kicking would be delayed longer during rehabilitation than in a MT strain to avoid a possibility of re-injury or tendon retraction [20]. Hence careful anatomical location and severity do have therapeutic and prognostic implications which guide the decision making process and RTS for each RF injury subgroup. The outcome based on intervention for each injury subcategory in the studies are summarized in figure 2.

### Myotendinous injuries

RF injury is common with kicking or sprinting [3, 17–19]. This was a consistent finding amongst the inclusions describing MT injuries. We found these to occur similarly across studies. Most kicking injuries resulted in tears on the MT area which need surgical intervention following failed conservative therapy [48, 58, 68, 69, 77]. The low certainty of evidence suggests that upon diagnosis of an MT tear surgical intervention may be warranted sooner to avoid prolonged RTS times. An MRI in this case may assist in evaluating the extent of soft-tissue damage that occurred with the injury to determine the appropriate intervention. Fermín Valera-Garrido *et al.* [78] through a combination of US-Guided PNE 48 hours after grade II MT junction injuries and a specific rehabilitation program achieved optimal injury repair. RTS was also within a short period (15.62 ± 1.80 days) and no re-injuries were reported on follow-up [78].

Athletes who sustained MT strains during sprinting may be treated successfully with conservative rehabilitation modalities [77]. However, insufficient detail on the conservative programmes renders it impossible to compare conservative approaches or replicate them. Lack of consistent reporting on outcomes (RTS and re-injury outcome) for MT injuries treated conservatively shows the lack of uniformity or evidence-guided approach with regards to the treatment of MT injuries.

In the surgical intervention studies on MT injuries, the mean RTS is 6 months post-intervention, and no re-injuries were documented in the first 12 months or longer follow-up. The incomplete reporting on outcome measures makes it difficult to establish the superiority of either intervention. A successful RTS time could be shortened by a proper evaluation and evidenced-based intervention guided by MRI imaging to reduce the delay to surgery time for those cases needing surgical intervention.

### Free tendon tear

The majority of FT tears to the RF are reported during kicking (63%) and involve the direct tendon (72%) [21, 43–45, 61, 62]. This is contrary to an earlier finding by Serner *et al.* [22] that a vast majority of RF injuries (94%) involved the proximal tendons, commonly the indirect tendon (56%) in comparison to the direct tendon insertion [22]. Tears may in some instances involve the direct and indirect tendons concurrently; [49, 63] the acetabular labrum [49] or the conjoint tendon [51]. MRI remains a key investigation tool for the evaluation of FT tears. Ultrasound imaging may also be used successfully, [63] and may be valuable in resource-limited settings. If clinically indicated, MRA (arthrogram) is shown to be helpful in the evaluation of intraarticular pathologies such as labral tears [49].

Most athletes that underwent conservative treatment were from the sprinting group (n = 10) [49, 61]. The conservative program was not outlined [61]. Surgical intervention mostly followed failed conservative treatment [21, 44]. Irmola *et al.* [21] reported a 3–4 cm retraction of the avulsed segment while Adler et al. [44] reported scar tissue intraoperatively. This highlights the aspect of a longer delay to surgery which could go up to 15 months hence contributing to a lengthened recovery and RTS period.

The consideration of treatment modality based on indication was shown to yield early surgical intervention in some studies such as Ueblacker *et al.* [51] and Garcıá *et al.* [45] who acutely performed surgical repair in FT tears. These indications were either a direct tendon avulsion, [45] > 2 cm tendon retraction [51] on MRI or the patient’s preference [62]. The lack of standardized protocols accounts for this heterogeneity of indications which may be explored in future studies for an evidence-based approach.

Both conservative and surgical interventions amongst the studies show a good outcome with little to no complications on long-term follow-ups. With the limited evidence, successful conservative treatment yielded a quicker RTS within 14.5 (4–36) weeks [43, 61, 63] over the surgical approach that reported 5 (2.5–9) months. However, RTS was found to be longer at 9 months when there was labral involvement; in this case from a free indirect tendon avulsion. In these athletes, none had a re-injury at 15 months follow-up [49]. The standard surgical approach involved re-anchoring the torn tendon to its origin (suture anchor repair) [21, 44, 45, 51, 62].

When surgical intervention was conducted acutely [45, 51] there was essentially no difference in the respective RTS time of athletes treated surgically and conservatively as both RTS in roughly 3 months post-intervention. More high yield studies will need to be conducted to substantiate this early finding and possible confounding factors. Nonetheless, the limited evidence suggests that surgical intervention remains a solution for athletes with a failed conservative treatment evidenced by no re-injuries on long-term follow-up postoperatively [21, 44].

With either intervention, there were no re-injuries noted in the follow-up periods ranging between 2 and 35 months with minimal complications. Irmola *et al.* [21] reported on only one athlete that sustained a lateral cutaneous nerve hypersensitivity on follow-up which resolved within a year.

### Anterior inferior iliac spine avulsion fracture

Most (90%) RF avulsion fractures occur at the AIIS in adolescent males from a kicking mechanism and were reported in soccer (29%) [46, 47, 49, 50, 52, 53, 55, 64–67, 73, 75, 76]. X-ray in this subgroup is shown to be effective as a primary imaging tool [49, 55–57, 65, 71, 73, 74, 76]. In some instances, computed tomography (CT) scan and MRI may play a role as in the cases presenting with features of extra-articular impingement [46, 52, 67].

Conservative treatment proved to be effective in most avulsion fractures with a mean RTS of 2.9 months in the studies that reported RTS [49, 53, 57, 64–66, 74, 76] compared to surgical interventions with a mean RTS of roughly 6 months. In either of the interventions, RTS may be longer with associated labral tears as is the case with FT tears. Most studies reporting on re-injury follow-up had no re-injuries between 12- and 24-months reviews. Hence neither surgical approach nor technique yielded superior outcomes.

The small number (n = 9) that needed surgical intervention were mostly from the kicking cohort (n = 7, 81%). Indications for operation range from non-union, [46, 49, 50, 67] mal-union [52, 54] and heterotrophic ossification [42, 47, 52]. In all these instances conservative treatment had failed. In some of these cases, there can be secondary impingement that warrants surgical correction [49, 52, 54, 67]. This is evidenced by a long delay to intervention ranging between 2 and 24 months after a trial of conservative treatment. Despite the delay, surgical treatment remains a viable option when conservative treatment fails as all athletes returned to sport and had no re-injuries on follow-up [49, 52, 54, 67]. The surgical approach can either be arthroscopic [42, 49, 54, 67] or open fixation, [46, 47, 50, 52, 55] and involves an ORIF or excision of the avulsed segment and suture anchor repair to AIIS origin. After failed conservative treatment there usually was fibrosis or exuberant scar tissue formation consistent with a longstanding injury.

### Practical application

**FIG. 3:**
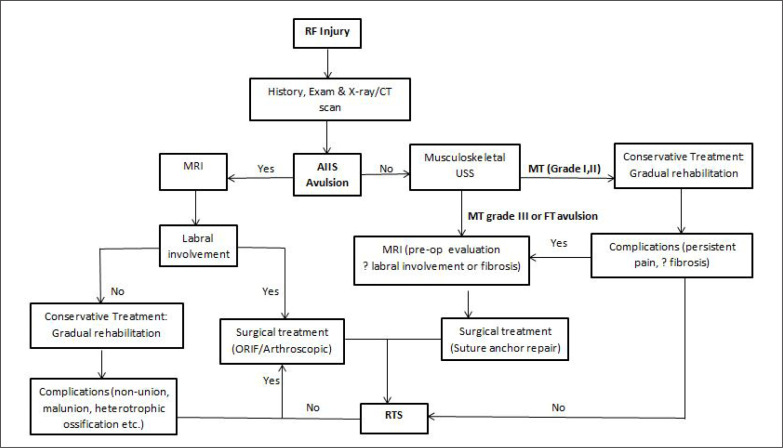
Rectus femoris (RF) injury treatment algorithm.

Based on low certainty of evidence; in practice careful evaluation of the injury with guided imaging are paramount to an anatomical diagnosis for a guided treatment plan with a timely RTS and less likelihood of complications as outlined in Figure 3. Future high-level research is warranted to validate these findings.

The assessment of the effectiveness of the intervention based on both RTS and re-injury outcomes is challenging as most studies did not report re-injury follow-up. Along with the low-level evidence of the included papers a comparison of the intervention-based outcome remains challenging. Conversely, for hamstring [33, 86–88] and Anterior Cruciate Ligament (ACL) [89–92] injuries extensive research has been done regarding surgical and conservative intervention with detailed rehabilitation plans. The relatively long RTS time of RF injuries warrant similar attention.

### Limitations

The major limitations in this systematic review consist of the low level of evidence of the included studies, no outline of the conservative intervention program in most papers and incomplete reporting of both the primary outcome measures namely RTS and re-injury follow-up evaluation. These factors along with the heterogeneity in reporting amongst the studies made it difficult to conduct a meta-analysis.

The reported studies have a limited number of athletes to allow clustering based on either the age groups or sporting code (team or individual sports) hence presents a challenge of confounding factors associated with the risk associated with individual or team sports and a wide age group [93, 94]. Regarding the GRADE (Grading of Recommendations, Assessment, Development and Evaluations) assessment [95, 96]. With the effectiveness question at hand and only case series/ reports in our inclusions, we were not able to provide evidence of effectiveness (causality). We could also not perform a summary table of findings as per GRADE standards but could only make comments about associations.

### Future Research

More research on the topic is needed regarding standardised treatment protocols, time to RTS and the incidence of re-injury. It is therefore proposed that future research should follow a form of multi-centre randomized controlled trials including a detailed conservative program and a standardized surgical program with long-term follow-up also determining RTS time and re-injury rate. Such research is required for all the different RF injury regions.

## CONCLUSIONS

Based on the low certainty of evidence we conclude that RF injury occurs mostly from kicking. This mechanism is associated with more complicated injuries amongst the different injury groups (MT, FT, AIIS avulsion injury). Kicking commonly leads to either a tear or avulsion at the FT and AIIS regions with or without a labral tear. With low certainty, it is suggested that successful conservative treatment provides a shortened RTS outcome amongst the injury regions. Surgical treatment also remains an option for failed conservative treatment of RF injuries across all the injury groups. The complicated cases are treated successfully through surgical means either primarily or after a failed course of rehabilitation. Primary surgical interventions seem to have similar recovery rates and low failure rates and can be considered in elite athletes where prolonged RTS can have far-reaching consequences. Conservative and surgical intervention in the different injury groups had limited to no complications evidenced by the lack of re-injuries on follow-up. With this low evidence synthesis, more high-level studies are strongly recommended to improve the evidence base for the treatment of this significant injury.

The authors confirm that the data supporting the findings of this study are available within the article [and/or] its supplementary materials.

## Figures and Tables

**Supplementary Table S1:** Search strategy

Search string	Database or further sources	Results	Date	Comments
rectus femoris AND injury AND running rectus femoris strain AND injury AND anterior inferior iliac spine	SPORTDISCUSS	147	2020-04-12	COMBINED RF “AND” AIIS Avulsion search

rectus femoris AND Injury AND Treatment “OR” Anterior inferior iliac spine AND Avulsion AND Treatment	MEDLINE	50	2020-04-14	RF “OR” AIIS Avulsion

((anterior inferior iliac spine avulsion AND treatment)) OR (Rectus femoris AND Injury AND Treatment AND outcome AND (“1979/01/01” [PDat]; “2020/12/31” [PDat]) AND Humans [Mesh])	PUBMED SEARCH	88	2020-04-14	AIIS avulsion “OR” RF Injury

RF = Rectus femoris; AIIS = Anterior inferior iliac spine

**Supplementary Table S2:** Methodological quality assessment outcome MS Excel file

**Supplementary Table S3:** Exclusions and Reasons

	Study	Reason for Exclusion
1	Knobloch *et al,* 2007	Full text not assessable, Article not in English (German)

2	Takahara *et al*	Full text not assessable, Article not in English (Japanese)

3	Kayipmaz *et al,* 2011	Full text not assessable, Article not in English (Turkish)

4	Langer & Selesnick, 2010	No Outcome: Return to Play outcome

5	Temple *et al,* 1998	Injury mechanism not outlined, 1 athlete with mechanism was > 45

6	R Zini *et al,* 2014	Injury mechanism not outlined

7	Sonnery-Cottet *et al,* 2017	Injury mechanism not clear: “eccentric mechanism”

8	Ali *et al,* 2015	No outcome. Only “Improvement” reported

9	Balius *et al,* 2009	Injury mechanism not outlined (“during football”)

10	Bartl *et al,* 1997	Full text not assessable, Article not in English (German)

11	Calderazzi *et al,* 2018	No primary data (systematic review)

12	Dean *et a,* 2016	No primary data (Technical report)

13	Eberbach *et al*, 2017	No primary data (systematic review)

14	Incedayi *et al,* 2014	Injury mechanism not outlined

15	Linni *et al*, 2000	Full text not assessable, Article not in English (German)

16	Novais *et al*, 2018	Mechanism not kicking or sprinting (direct trauma)

17	Pierannunzii *et al,* 2010	Mechanism not kicking or sprinting (“active extension of hip from flexed position”)

18	Schwobel *et al,* 1985	Full text not assessable, Article not in English (German)

19	Sinikumpu *et al,* 2018	Injury mechanism not outlined

20	Stancak, 2016	Full text not assessable, Article not in English (Czech)

21	Stancak, 2016	Full text not assessable, Article not in English (Czech)

22	Sulko *et al,* 2010	Full text not assessable, Article not in English (Polish)

23	Weber *et al*, 2010	Mechanism not kicking or sprinting (Forced knee flexion during fall)

24	Yun *et al*, 2009	Age out of range (52 years)

25	Zini *et al,* 2018	Injury mechanism not outlined

26	Yildiz *et al,* 2003	No full-text, Abstract not sufficient

27	Jethwa *et al,* 2020	Age out of range (53 years old)

28	Cho *et al,* 2020	Outcome (RTS or re-injury) not outlined – reports improvement in hip range of motion and athlete walking at week 3

29	Maalouly et al, 2020	Mechanism not kicking or sprinting – Hip hyperextension

30	Sinikumpu, 2018	Mechanism not outlined

31	Lempainen, 2018	Mechanism not kicking or sprinting – sudden pain during hip extension or forceful flexion

32	Kayani *et al,*2021	No outcome

33	Santos et al, 2021	No intervention or outcome reported
